# Cladribine and Fludarabine Nucleoside Change the Levels of CD Antigens on B-Lymphoproliferative Disorders

**DOI:** 10.1155/2010/964251

**Published:** 2010-05-05

**Authors:** Carlos Cassano, Swetlana Mactier, Stephen P. Mulligan, Larissa Belov, Pauline Huang, Richard I. Christopherson

**Affiliations:** ^1^School of Molecular and Microbial Biosciences, University of Sydney, Sydney, NSW 2006, Australia; ^2^Department of Haematology and Transfusion Medicine, Royal North Shore Hospital, St. Leonards, NSW 2065, Australia

## Abstract

The purine analogs, fludarabine nucleoside (FdA), and cladribine (CdA) (1 *μ*M, 24 hours), significantly changed the levels of some surface antigens on the human B-cell lines MEC2 and Raji. Changes in the surface proteins were identified using a Cluster of Differentiation (CD) antibody microarray that captures live cells and confirmed by flow cytometry. For Raji cells, CdA up-regulated CD10, CD54, CD80, and CD86, with repression of CD22, while FdA up-regulated CD20, CD54, CD80, CD86 and CD95. For MEC2 cells, CdA up-regulated CD11a, CD20, CD43, CD45, CD52, CD54, CD62L, CD80, CD86, and CD95, but FdA had no effect. Up-regulation of particular CD antigens induced on a B-cell lymphoproliferative disorder by a purine analog could provide targets for therapeutic antibodies with synergistic cell killing.

## 1. Introduction

Purine nucleoside analogs are effective in the treatment of some B-lymphoproliferative disorders (B-LPDs). Fludarabine (FdAMP, 9-*β*-D-arabinofuranosyl-2-fluoroadenine 5′-monophosphate) induces cell cycle arrest and apoptosis [1] and is used to treat patients with chronic lymphocytic leukemia (CLL) and indolent non-Hodgkins lymphoma (NHL) [2–4]. Cladribine (CdA, 2-chloro-2′-deoxyadenosine) is effective against CLL, low-grade NHL, and hairy cell leukemia (HCL) [5]. FdAMP is administered as the nucleoside 5′-monophosphate derivative and is hydrolyzed outside the cell to fludarabine nucleoside (FdA) then transported and phosphorylated inside the cell (FdA → FdAMP → FdADP → FdATP). CdA is administered as the nucleoside derivative and converted to the triphosphate (CdATP). FdATP and CdATP both inhibit ribonucleotide reductase, and at higher concentrations they inhibit DNA and RNA synthesis [3, 6] and induce apoptosis [7]. The main side-effect of these purine analogs is myelosuppression leading to opportunistic infections [8, 9]. 

Cell surface molecules are classified as Cluster of Differentiation (CD) antigens (Human Cell Differentiation Molecules-HCDMs, http://www.hlda8.org/) and have a variety of functions such as cytokine and growth factor receptors, transporters, ion channels, cell-cell interactions, and adhesion molecules [10]. CD antigens are potential targets for treatment of B-LPD with therapeutic antibodies. For example, antibodies against CD20 (rituximab, Mabthera), CD52 (alemtuzumab, Campath-1H), and CD80 (galiximab) are effective for treatment of some B-LPDs such as CLL and NHL [11, 12]. Immunotoxins such as BL22 directed against CD22 may be useful in the treatment of CLL, NHL and HCL [13]. Radioimmunoconjugates, such as ^131^I-tositumomab (Bexxar) and ^90^Y-ibritumomab tiuxetan (Zevalin), directed against CD20 are effective for treatment of NHL [14]. 

Recent clinical trials support combination of a purine nucleoside analog with a therapeutic antibody for treatment of CLL and NHL [3]. Combination therapy with rituximab and FdAMP prolongs progression-free survival of patients with previously untreated CLL [15]. FdAMP, cyclophosphamide, and rituximab (FCR) produce a high rate of complete remissions in untreated CLL [16]. FdAMP and alemtuzumab are active against CLL that is refractory to either drug alone [17]. The combination of CdA with rituximab is also effective against CLL and indolent lymphoma [18]. These results indicate synergy between purine analogs and therapeutic antibodies. 

Antibody microarray technology is a powerful tool in proteomics, target discovery, and differential analysis. A CD antibody microarray (DotScan^TM^) has been developed in our laboratory that enables extensive surface profiles of leukocytes to be obtained in a single, simple assay. DotScan^TM^ has been used to determine immunophenotypic changes on human HL60 leukemia cells during differentiation [19, 20]. In the present work, we have used the microarray to identify CD antigens whose levels change on the human B-lymphoid cell lines MEC2 (derived from CLL) and Raji (from Burkitt's lymphoma) treated with CdA or FdA, with subsequent quantitative analysis of those antigens by flow cytometry. The results show that CdA (1 *μ*M, 24 hours) changes the surface protein profiles of MEC2 and Raji cells and FdA (1 *μ*M, 24 hours) changes the profile of Raji but not MEC2. Up-regulation of particular CD antigens induced on a B-LPD by a purine analog could provide targets for combined treatment with purine analogs and therapeutic antibodies, directing cytotoxic activity specifically against the neoplastic cells.

## 2. Materials and Methods

### 2.1. Reagents

RPMI 1640 medium (Hepes modification), FdA, and Trypan blue were purchased from Sigma Aldrich (Castle Hill, Australia). CdA was a kind of gift from the National Cancer Institute (Bethesda, USA). Gentamicin (50 mg gentamicin sulfate/mL), L-glutamine (200 mM), and fetal calf serum were from Invitrogen (Mulgrave, Australia). The Annexin V-PE Apoptosis Detection Kit I was from BD Pharmingen (North Ryde, Australia).

### 2.2. Cell Lines and Their Culture

MEC2 cells (derived from a B-cell CLL) were a kind of gift from Dr. Federico Caligaris-Cappio, (University of Torino, Italy; [21]). Raji cells (from a B-cell Burkitt's lymphoma) were from the American Type Culture Collection (Manassas, USA; [22]). MEC2 and Raji cells were grown in Hepes-buffered RPMI 1640 medium with 10% (v/v) fetal calf serum, 50 *μ*g/mL gentamicin, and 2 mM L-glutamine at 37°C in a nonhumidified atmosphere without CO_2_. Cultures of MEC2 and Raji at a density of 3 × 10^5^ cells/mL were treated with CdA or FdA (1 *μ*M, 24 hours) and harvested for surface profiling of CD antigens.

### 2.3. Apoptosis Detection

Externalization of phosphatidylserine (PS) on cells during apoptosis was quantified by Annexin V-PE binding, while the loss of cell membrane integrity indicating dead cells was demonstrated by binding of 7-amino-actinomycin D (7-AAD) to DNA. Briefly, cells were washed twice with PBS and resuspended at a density of 10^6^ cells/mL in binding buffer (10 mM Hepes pH 7.4, 140 mM NaCl, and 2.5 mM CaCl_2_). An aliquot of the suspension (50 *μ*L, 0.5 × 10^5^ cells) was incubated with 2.5 *μ*L of Annexin V-PE and 2.5 *μ*L of 7-AAD for 15 minutes at room temperature. Cells were then resuspended in 450 *μ*L of binding buffer and analyzed with an FACScan flow cytometer (Becton Dickinson, San Jose, USA) using CellQuest software.

### 2.4. Profiling of Cells on CD Antibody Microarrays

DotScan^TM^ microarrays were obtained from Medsaic (Eveleigh, Australia). Capture of cells on 82-dot CD antibody microarrays, imaging, and data analysis have been described [23]. Dot patterns of cells captured on CD antibody microarrays were imaged and analyzed using a scanner and software from Medsaic [23]. Data were normalized to a maximum 8-bit dot intensity value of 256. Experiments to determine the effects of CdA or FdA (1 *μ*M, 24 hours) on the immunophenotypes of cells were performed in triplicate. For each cell line, three cell cultures were treated with CdA or FdA dissolved in dimethylformamide, while the three control cultures were treated with the corresponding amount of dimethylformamide. Data are expressed as means with standard errors. 

### 2.5. Flow Cytometry of Cells

CD antigens whose levels changed on Raji and MEC2 cells using DotScan^TM^ were also analyzed quantitatively by flow cytometry using FITC- or PE-conjugated monoclonal antibodies and standard procedures. Fluorescently-labelled monoclonal antibodies were obtained from Becton-Dickinson Pharmingen (North Ryde, Australia), IMM, Immunotech, Beckman Coulter (Gladesville, Australia), and CALTAG, Invitrogen (Mount Waverley, Australia). Samples were analyzed using an FACScalibur flow cytometer (Becton Dickinson, San Jose, USA) with a 488-nm air-cooled argon-ion laser, running CellQuest Pro software. Fluorescence detection used logarithmic amplification (FL1 (green) = 530/30 nm; FL2 (red) = 585/40 nm). Quantitative data are the averages of 3 determinations with variance expressed as *P*-values.

## 3. Results

### 3.1. Effects of Purine Analogs on Cell Growth and Viability

**Figure 1:**
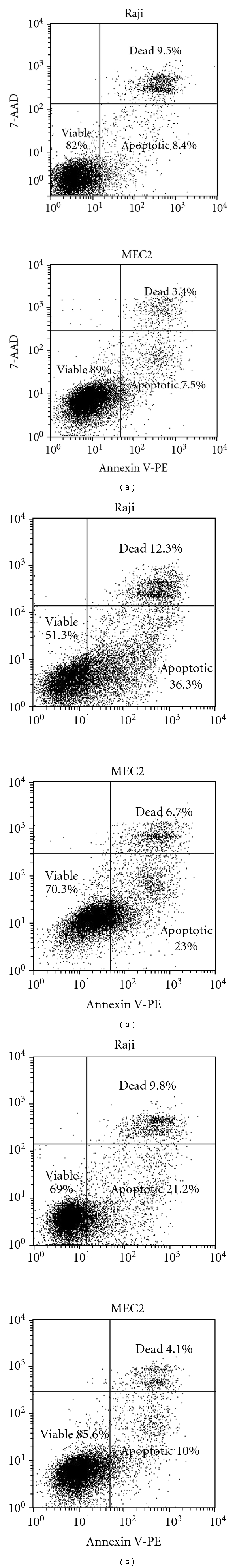
Induction of apoptosis of Raji and MEC2 cells by purine analogs. Samples of cells were taken at the indicated times, stained with Annexin V-PE and 7-AAD, and analyzed by flow cytometry as described in Materials and Methods. (a) Control, (b) CdA (1 *μ*M, 24 hours), and (c) FdA (1 *μ*M, 24 hours).

Cell viability was assessed by Trypan blue exclusion. CdA (1 *μ*M) arrested growth of Raji and MEC2 cells, and FdA (1 *μ*M) arrested Raji cells but not MEC2 cells (data not shown). Apoptotic and dead cells were quantified using Annexin V-PE and 7-AAD with flow cytometry (Figure 1). CdA (1 *μ*M, 24 hours) induced for Raji: apoptosis (control 8.4%, + CdA 36.3%) and cell death (control 9.5%, + CdA 12.3%), for MEC2 cells: apoptosis (control 7.5%, + CdA 23%) and cell death (control 3.4%, + CdA 6.7%). FdA (1 *μ*M, 24 hours) induced for Raji: apoptosis (control 8.4%, + FdA 21.2%) and cell death (control 9.5%, + FdA 9.8%). FdA (1 *μ*M, 24 hours) had no significant effect on MEC2: apoptosis (control 7.5%, + FdA 10%) and cell death (control 3.4%, + FdA 4.1%). 

### 3.2. Effects of Purine Analogs on Cell Surface Profiles Using Microarrays

**Figure 2:**
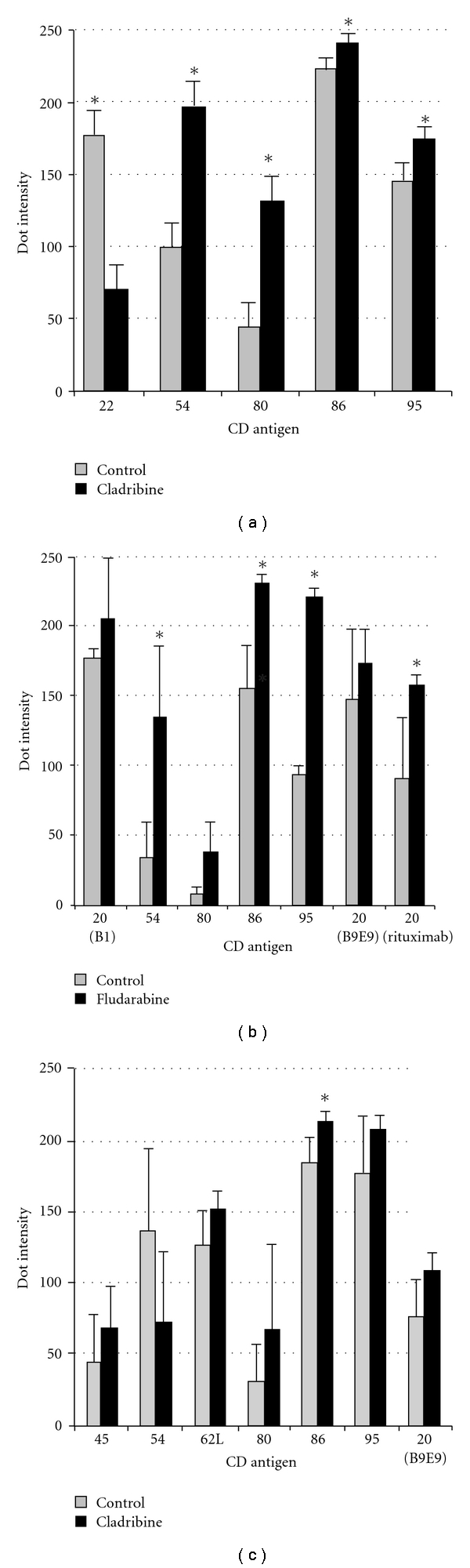
Expression of CD antigens on Raji and MEC2 cells treated with purine analogs (1 *μ*M, 24 hours) analyzed using microarrays. (a) Raji + CdA, (b) Raji + FdA, and (c) MEC2 + CdA. Expression profiles were generated from the dot patterns of cells captured on microarrays; control (grey bars), drug-treated (black bars). CD antigens are labelled on the abscissa, average dot intensities on an 8-bit greyness scale (1–256) on the ordinate. Mean dot intensities are plotted with standard error bars (*n* = 3). Asterisks indicate significant (*P* < .05) changes.

CD antibody microarrays were used to screen for immunophenotypic changes on Raji and MEC2 cells induced by CdA or FdA (1 *μ*M, 24 hours). The expression profiles in Figure 2 show mean cell binding densities from analysis of control and drug-treated cultures (*n* = 3). For Raji cells, CdA (1 *μ*M, 24 hours) increased levels of CD54, CD80, CD86, and CD95, while CD22 was repressed (Figure 2(a)). FdA (1 *μ*M, 24 hours) increased levels of CD20 (rituximab), CD54, CD80, CD86, and CD95 (Figure 2(b)). Although an increase in CD20 was observed with two other clones of CD20 antibody (B1, B9E9), these changes were not significant (*P* > .05). For MEC2 cells, CdA increased CD20 (B9E9), CD45, CD62L, CD80, CD86, CD95, while CD54 was repressed (Figure 2(c)). The changes for MEC2 with CdA were consistent, but only CD86 up-regulation was significant (*P* < .05). DotScan^TM^ microarrays provide extensive surface profiles of CD antigens that are semiquantitative. Flow cytometry was used to confirm and quantify levels of CD antigens. 

### 3.3. Effects of Purine Analogs on Surface Antigens Using Flow Cytometry

**Figure 3:**
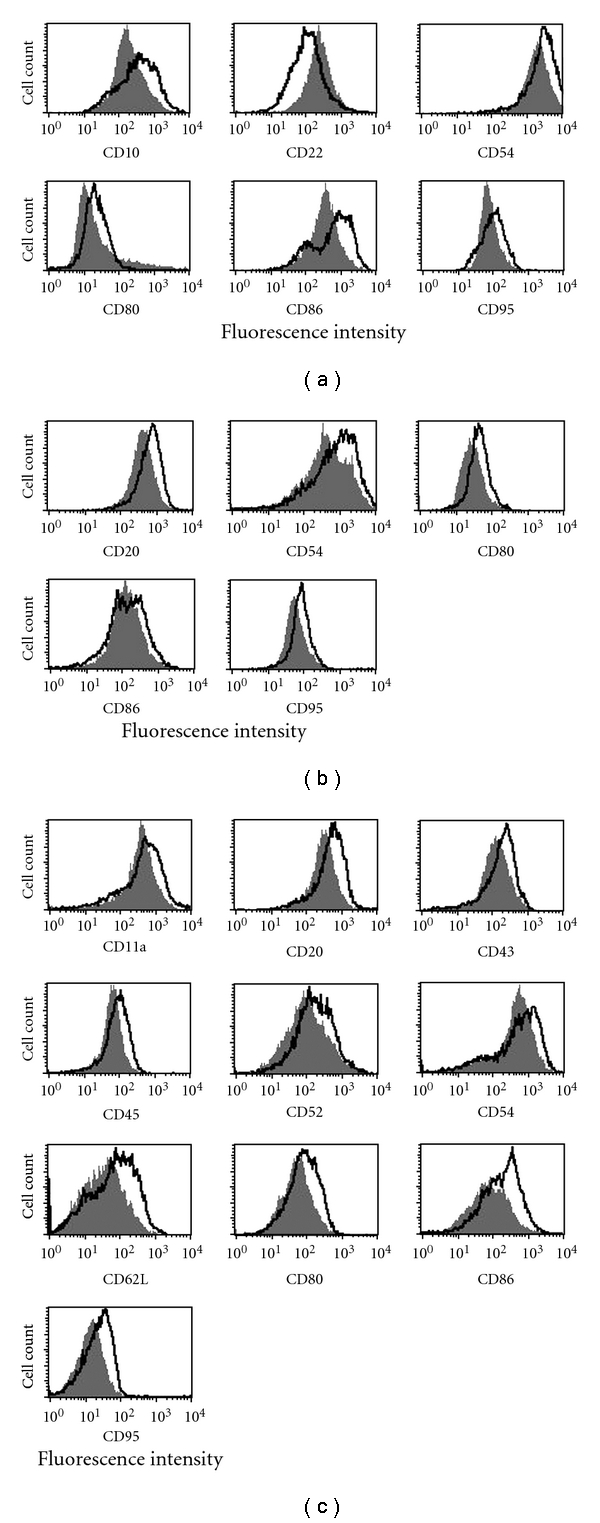
Expression of CD antigens on Raji and MEC2 cells treated with purine analogs (1 *μ*M, 24 hours) analyzed using flow cytometry. (a) Raji + CdA, (b) Raji + FdA, and (c) MEC2 + CdA. Raji and MEC2 cells were exposed to CdA or FdA (1 *μ*M, 24 hours), incubated with FITC- or PE-conjugated antibodies against CD antigens, and analyzed by flow cytometry. The grey area represents untreated cells; the black outline is cells exposed to a purine analog. The figures are representative of 3 technical replicates. A shift in the median fluorescence intensity indicates up- or down-regulation of an antigen. Differential abundances of these CD antigens are quantified in Table 1.

For confirmation, MEC2 and Raji cells were treated with CdA or FdA (1 *μ*M, 24 hours), and CD antigens of interest were analyzed by flow cytometry. CellQuest Pro software (Becton Dickinson, San Jose, USA) was used to overlay the fluorescence histograms and obtain the median fluorescence intensities for calculation of the fold-changes of CD antigen expression (drug-treated)/(control).  Figure 3 shows overlays of fluorescence histograms of control (grey) and drug-treated (black outline) samples. These results confirm that CdA (1 *μ*M, 24 hours) induced increases on Raji cells of CD54, CD80, CD86, and CD95, and CD22 was decreased (Figure 3(a)). In addition, increased CD10 was detected on Raji cells treated with CdA. Raji cells treated with FdA (1 *μ*M, 24 hours) showed increased CD20, CD54, CD80, CD86, and CD95 (Figure 3(b)). MEC2 cells treated with CdA (1 *μ*M, 24 hours) showed increased CD20, CD45, CD62L, CD80, CD86, and CD95. Increased CD11a, CD43, CD52, and CD54 were also detected by flow cytometry but not microarrays. No changes in CD antigen expression were observed for MEC2 cells treated with FdA (data not shown).  Table 1 summarizes the flow cytometric data for Raji and MEC2 cells treated with these purine analogs (Figure 3). 

## 4. Discussion

CdA and FdAMP are effective for treatment of CLL and low-grade NHL [24]. Recent studies indicate that purine analogs administered in combination with other drugs and/or therapeutic antibodies (rituximab and alemtuzumab) may produce higher response rates including complete remissions [25]. Proteomic analysis of Raji cells treated with FdA using two-dimensional gel electrophoresis and mass spectrometry has identified new nuclear regulatory proteins that may participate in FdA-induced apoptosis. Analysis of cytosolic and mitochondrial proteins has identified 25 proteins that differ by more than 2-fold between control cultures and purine analog-treated Raji cells (Mactier, Henrich, and Christopherson, manuscript submitted). These proteins provide further insight into the mechanisms of action of purine analogs and may represent new therapeutic targets. Antibody microarray-based technology is a promising tool in the field of proteomics, in which one of the important applications is to compare proteomic profiles of untreated and drug-treated cancer cells to identify potential drug targets as well as further elucidate mechanisms of drug treatment. In the present study, DotScan^TM^ antibody microarrays were used to screen for changes induced by purine analogs on the surface of leukemia and lymphoma cell lines. CdA and FdA induced up-regulation of a number of CD antigens on MEC2 and Raji cells that could be targets for synergistic treatment with therapeutic antibodies. 

CD10, a neutral endopeptidase and integral membrane protein whose expression correlates with apoptosis [26, 27], was induced 2.4-fold on Raji cells treated with CdA (Table 1). CD54 (intercellular adhesion molecule-1, ICAM-1), a member of the immunoglobulin superfamily of adhesion molecules, was up-regulated 1.4- to 1.8-fold on Raji cells treated with CdA or FdA and on MEC2 cells treated with CdA (Table 1). CD11a (lymphocyte function-associated antigen 1, LFA-1 integrin) increased 1.4-fold on MEC2 cells treated with CdA (Table 1). When T cells encounter antigen-presenting cells (APCs), cell-cell contact is established between LFA-1 (CD11a) and ICAM-1 (CD54). CD20 was induced 1.6-fold on MEC2 cells treated with CdA and 1.5-fold on Raji cells treated with FdA (Table 1). CD20, a B-cell antigen, is a calcium channel that regulates cell-cycle progression and proliferation. CD20 is expressed at lower levels on B-CLL compared with normal B-cells and other B-LPD [28]. Van Meerten et al. [29] showed that increased CD20 enhances antibody-dependent cellular cytotoxicity (ADCC) and complement dependent cytotoxicity (CDC). Combination therapy using CdA or FdAMP with rituximab is clinically synergistic against CLL and lymphomas [18, 30, 31]. The up-regulation of CD20 observed (Table 1) may provide an explanation for this synergy. 

CD22, a regulator of the B-cell receptor (BCR), was repressed 0.51-fold on Raji cells treated with CdA (Table 1). CD22 is a B-cell-specific sialoglycoprotein of the immunoglobulin superfamily commonly expressed on NHL [32] involved with cellular adhesion and homing and regulation of B-cell activation. Monoclonal antibodies against CD22 that block ligand binding inhibit normal and malignant B-cell survival [33]. Repression of CD22 on Raji cells by CdA may reduce CD22 signaling, decrease proliferation, and promote apoptosis. CD43 was up-regulated 1.5-fold on MEC2 cells treated with CdA (Table 1). CD43 participates in signaling and activation of T lymphocytes [34]. Overexpression of CD43 induces accumulation of p53 [35], while monoclonal antibodies binding to CD43 may induce apoptosis [36–38]. 

CdA induced CD52 1.6-fold on MEC2 cells (Table 1). Alemtuzumab (Campath-1H), a therapeutic antibody against CD52, induces CDC and ADCC and is effective for treatment of CLL [25]. It was shown that alemtuzumab in combination with a purine nucleoside analog may be effective for treatment of refractory CLL [17]. CD62L (L-selectin, LECAM-1) is up-regulated 2-fold on MEC2 cells treated with CdA (Table 1). The expression of adhesion molecules may alter the mobility and homing of CLL cells and the immune response to CLL. The level of CD62L on B-CLL is usually less than half that of normal lymphocytes, possibly contributing to accumulation of B-CLL in peripheral blood [39]. 

CD80 and CD86 are up-regulated 1.4- to 2.5-fold on Raji and MEC2 cells treated with CdA or FdA (Table 1). These coregulators of T cell activation induce proliferation, cytokine production, and effector functions. Up-regulation of CD80 and CD86 may enhance a T cell response to CLL and lymphoma [40]. CD80 may also play a role in regulation of normal and malignant B-cells [41] and may be constitutively expressed on NHL, making it an attractive target for lymphoma therapy [42]. Galiximab is a therapeutic antibody directed against CD80 that has been used to treat NHL [11, 43]. CD95 is up-regulated 1.2- to 1.5-fold on Raji and MEC2 cells treated with CdA or FdA (Table 1). The Fas (CD95)/Fas ligand (CD178) system has a potential role in cytotoxic killing of some leukemias [44]. CdA-induced apoptosis may be triggered by the expression of Fas and Fas-L [45], and FdA induces Fas and Fas-L mRNAs [46]. The enhanced expression of CD95 on CLL cells treated with CdA or FdA could promote cell killing. 

While CdA and FdA are structurally similar, there are some differences in their effects. In this study, CdA induced immunophenotypic changes and apoptosis in the CLL-derived cell line MEC2 that is resistant to FdA. A recent clinical trial showed prolongation of therapeutic response with CdA compared to FdAMP [47] suggesting that CdA should be further investigated for treatment of CLL. Surface molecules up-regulated by a purine analog could be targeted with a therapeutic antibody providing synergy and more effective treatment. Our results may explain the clinical synergy between FdA and rituximab. CdA and FdA upregulate CD antigens on Raji and MEC2 cells that are targets for therapeutic antibodies such as rituximab (anti-CD20), alemtuzumab (anti-CD52), and galiximab (anti-CD80). CD10, CD11a, CD43, CD54, CD62L, and CD86, also up-regulated by FdA or CdA (Figures 2 and 3, Table 1), are potential targets for development of therapeutic antibodies, for drug-antibody combination therapy. Preliminary experiments with primary cells from CLL patients showed that CdA or FdA (1 *μ*M, 48 hours) induces increases in CD20, CD54, and CD95 (not shown). 

In summary, microarray analysis of Raji cells treated with purine analogs revealed significant changes (*P* < .05) in expression levels of several cell surface antigens, that were confirmed by flow cytometry (Figures 2 and 3, Table 1). Flow cytometry also revealed significant CdA-induced increases in cell surface antigens on MEC2 cells (*P* < .05, Figure 3, Table 1), but not all of these changes were significant by microarray although the same antibody clones were used. The differences between microarray and flow cytometry results for CdA-treated MEC2 cells are not fully understood. Flow cytometry uses soluble fluorescent antibodies, which have greater flexibility and freedom of movement than antibodies immobilized on a nitrocellulose surface and therefore have easier access to cell surface antigens, with fluorescence intensity directly reflecting antigen expression levels. Cell viability is not essential for flow cytometry, while cell capture on antibody dots depends not only on the interaction of cell surface antigens with immobilized antibodies but also on energy-dependent processes required for robust attachment of antibody-bound cells to the nitrocellulose surface; damaged or dead cells bind weakly and are easily washed off during washing and fixation steps. The tendency of MEC2 cells to cluster rapidly in suspension may, at least in part, account for greater variability in replicate dot intensities for MEC2 compared to Raji (Figure 2(c) versus 2(a)), because cell clusters are less firmly bound to antibody dots than single cells due to reduced interaction of clustered cells with the immobilized antibodies. 

DotScan^TM^ microarrays provide a powerful screening tool for the detection of drug-induced modulations of cell surface antigens and could be used to screen patient samples to identify other potential synergistic drug-antibody combinations for CLL and other B-LPDs. Microarray-based proteomic analysis should also enable rational development of chemoimmunotherapy for less common disease entities and subsets and enable tailored treatment for individual patients.

## Figures and Tables

**Table 1:** Differential abundance of CD antigens on Raji and MEC2 cells after treatment with CdA or FdA assessed by flow cytometry.

	Raji + CdA		Raji + FdA		MEC2 + CdA	
CD antigen	Fold change^1^	*P*-value^2^	Fold change	*P*-value	Fold change	*P*-value
CD10	**2.4**	1 × 10^−3^	No change		No change	
CD11a	n.d.		n.d.		**1.4**	1.6 × 10^−4^
CD20	No change		**1.5**	6.5 × 10^−2^	**1.6**	2 × 10^−6^
CD22	**0.51**	2 × 10^−6^	No change		No change	
CD43	n.d.		n.d.		**1.5**	1 × 10^−5^
CD45	n.d.		n.d.		**1.3**	1.6 × 10^−2^
CD52	No change		No change		**1.6**	2.6 × 10^−3^
CD54	**1.7**	7 × 10^−4^	**1.8**	1.7 × 10^−2^	**1.4**	4 × 10^−3^
CD62L	n.d.		n.d.		**2.0**	3 × 10^−6^
CD80	**1.5**	1 × 10^−3^	**1.5**	5 × 10^−5^	**1.5**	2 × 10^−5^
CD86	**1.9**	3 × 10^−5^	**1.4**	1.2 × 10^−4^	**2.5**	1 × 10^−4^
CD95	**1.2**	8 × 10^−5^	**1.4**	1.1 × 10^−4^	**1.5**	2.8 × 10^−3^

^1^Fold change calculated by dividing the median fluorescence intensity for the drug-treated sample by that for the control (Figure 3).

^2^ *P*-value calculated using the students' *t*-test (*n* = 3).

n.d.: CD antigen not detected.

## Abbreviations

B-LPDs: B-lymphoproliferative disorders
CdA: Cladribine
FdA: Fludarabine nucleoside
FdAMP: Fludarabine.
